# The New Diagnosis-Related Group Reimbursement System and Laboratory Test Quality in Korea: Analysis of External Quality Assessment Results

**DOI:** 10.3390/healthcare8020127

**Published:** 2020-05-07

**Authors:** Sollip Kim, Yeo-Min Yun, Hyeongsu Kim, Tae-Hyun Um, Jeonghyun Chang, Hojin Jeong, Kun Sei Lee, Sail Chun, Yong-Jun Choi, Jae-Hyeok Heo, Tae-Hwa Han

**Affiliations:** 1 Department of Laboratory Medicine, Inje University, Ilsan Paik Hospital, Goyang 10380, Korea; lalacopine@gmail.com (S.K.); uthmd@hanmail.net (T.-H.U.); azacsss@naver.com (J.C.); 2 Department of Laboratory Medicine, School of Medicine, Konkuk University, Seoul 05030, Korea; 3 Department of Preventive Medicine, School of Medicine, Konkuk University, Seoul 05030, Korea; wyntme00@naver.com (H.J.); kunsei.lee@kku.ac.kr (K.S.L.); 4 Department of Laboratory Medicine, University of Ulsan College of Medicine and Asan Medical Center, Seoul 05505, Korea; sailchun@amc.seoul.kr; 5 Department of Social and Preventive Medicine, Health Services Research Center, College of Medicine, Hallym University, Chuncheon 24252, Korea; ychoi@hallym.ac.kr; 6 Department of Neurology, Seoul Medical Center, Seoul 02053, Korea; drjae93@gmail.com; 7 Health IT Center, College of Medicine, Yonsei University, Seoul 03722, Korea; taehwa.han@gmail.com

**Keywords:** diagnosis-related group, external quality assessment, quality of laboratory testing

## Abstract

Korea introduced a new diagnosis-related group (NDRG), which is a mixed-bundle reimbursement system. We evaluated the effects of NDRGs on laboratory test quality by analyzing data over three years (2016–2018) from the Korean Association of External Quality Assessment Service (KEQAS). A total of 42 NDRG-participating hospitals (CASE), 84 non-participating similar size-hospitals (CON-1), and 42 tertiary hospitals (CON-2) were included. We assumed the proportion of KEQAS results with a larger than 2 standard deviation index (SDI) to be a bad laboratory quality marker (BLQM). CASE BLQMs were lower than CON-1 BLQMs for more than 2 years in alkaline phosphatase (ALP), alanine aminotransferase (ALT), chloride, glucose, sodium, and total protein, and higher in creatinine. CASE BLQMs were higher than CON-2 BLQMs for more than 2 years in ALP, chloride, creatinine, glucose, lactate dehydrogenase (LDH), phosphorus, potassium, sodium, total calcium, total cholesterol, triglyceride, and uric acid. Mean SDIs for general chemistry tests were not significantly different depending on NDRG participation. However, the NDRG is currently a pilot program that compensates the amount of each institution’s reimbursement based on the fee-for-service system, and most participants were public hospitals. Thus, the effects of NDRGs on laboratory test quality should be re-evaluated after the NDRG program has stabilized and more private hospitals are participating.

## 1. Introduction

Increasing healthcare costs are a major concern in modern medicine. To reduce high medical costs and avoid unnecessary overtreatment, many countries have adopted diagnosis-related groups (DRGs) for reimbursements [1]. DRGs allow hospitals to receive fixed payment for the number and mix of patients they treat [2]. Accordingly, DRGs allow hospitals to reduce their costs per patient by cutting down the hospitalization period or intensity of services [3]. This generates conflicts of interest among physicians, mainly in terms of commitment to patient interests on the one hand and consideration of the hospital’s economic interests on the other [4]. DRGs bear inherent risks of potential harm to patients by quality of health services to reduce the cost of healthcare. Previous studies showed that implementing DRGs decreased the duration of stay and out-of-pocket costs [5] as well as significantly reducing examination fees relative to fee-for-service (FFS) [6].

The compulsory national health insurance program of Korea was originally based on an FFS system [7]. After starting a pilot program in 1997 and the voluntary period starting in 2002, Korea implemented a mandatory DRG-based payment system for seven groups of specific operations/diseases in inpatient care on 1 July 2012 [8]. However, the DRG design was difficult to extend, due to the rigidity of the model [9]. Therefore, the new DRG (NDRG), a mixed model that combines the existing DRG system with FFS, began in April 2009, as a pilot program for 20 disease groups with three public hospitals. Under the NDRG system, basic services such as hospitalization and tests, general procedures, and drug treatments are grouped into DRG-based payment, but doctors’ operations and procedures taken during hospitalization are not included into DRG-based payment and are separately reimbursed by the number of procedures. NDRGs expanded significantly in 2012 in terms of the number of disease groups and the number of participating hospitals; however, as it is still a pilot program, only 42 public hospitals covering 559 disease groups had participated up to 31 July 2018. As the system is still in the “introduction” period [9,10], there have been little data on the effects of NDRGs [11].

Laboratory tests are a large part of modern medicine. Nearly all inpatients, about half of patients in the emergency department, and nearly one-third of outpatients had laboratory results during their visit to the healthcare center [12], comprising a small portion (5–6%) of the total cost of healthcare [5,6]. Since poor laboratory results can significantly impact patients’ care, monitoring and improving the quality of laboratory tests is crucial [13]. In order to improve the quality of testing, the clinical laboratory in Korea conducts quality control internally, voluntarily participates in the external quality assessment (EQA) program, and also voluntarily receives a laboratory accreditation through external on-site inspection by an independent third party every one or two years. Even though participation in EQA and laboratory accreditation were not mandatory, hospitals receive a certain percentage of quality incentives based on clinical pathologist recruiting, EQA scores, and laboratory accreditation scores while they are in the FFS reimbursement system [14]. However, this quality incentive is only indirectly reflected in the NDRG through an adjustment factor, so the effect of incentives is not very noticeable.

We hypothesized that the introduction of the NDRG system could negatively affect the quality of laboratory tests because the NDRG is a modified DRG. Although there was a recent report regarding significantly reduced examination fees after the introduction of DRG [6], there have been no reports about the quality of laboratory tests after the introduction of DRGs or NDRGs. Thus, in this study, we aimed to evaluate the effects of NDRGs on the quality of laboratory tests by analyzing EQA results as a quality marker.

## 2. Materials and Methods

### 2.1. Study Subjects

Medical institutions in Korea are classified as clinics, (small-to-medium) hospitals, long-term care hospitals, general hospitals, and tertiary hospitals according to the Medical Service Act [15]. Since the term “hospital” is also a generic noun that includes several types of hospitals, we decided to use the Korean Medical Service Act’s term “hospitals” indicating “small-to-medium hospitals” to avoid ambiguity in this article. Hospitals should have more than 30 beds. General hospitals should have more than 100 beds with seven or more predefined medical departments (in case of general hospitals with 100–300 beds) or nine or more medical departments (in case of general hospitals with >300 beds) [15]. Tertiary hospitals should have at least 20 medical departments.

A total of 42 hospitals that participated in NDRGs before August 2018 were included in the study group (CASE). Additionally, 84 hospitals that did not participate in the NDRG system in July 2018 were selected for one control group (CON-1) by 1:2 matching based on number of hospital beds, and 42 tertiary hospitals not participating NDRGs were designated as another control group (CON-2) for reference comparison. The CASE group consisted of 35 public hospitals and seven private hospitals (33 general hospitals, eight small-to-medium hospitals, and one government institution). CON-1 consisted of all private hospitals (70 general hospitals and 14 small-to-medium hospitals). CON-2 consisted of 12 public hospitals and 30 private hospitals.

### 2.2. Data Source

The EQA results of the Korean Association of External Quality Assessment Service (KEQAS) database over three years (2016–2018) were analyzed for clinical laboratories, which were included as study subjects. EQA is a widely accepted method for evaluating clinical laboratories and enhancing their performance [16]. KEQAS was launched in 1976 and is the nation’s top authorized EQA provider for standardization and quality management of the laboratory tests of domestic hospitals. KEQAS was accredited as an EQA provider by International Organization for Standardization/International Electrotechnical Commission (ISO/IEC) 17043 in 2015. A total of 1862 hospitals participated in KEQAS in 2018, which correspond to approximately 49.1% of hospitals (including small-to-medium hospitals, general hospitals, and tertiary hospitals) that submit health insurance claims for laboratory testing in Korea [17]. 

### 2.3. Performance of General Chemistry Tests

The following 19 test items included in general chemistry tests were analyzed: albumin, alkaline phosphatase (ALP), alanine aminotransferase (ALT), aspartate transaminase (AST), blood urea nitrogen (BUN), chloride, creatinine, γ-glutamyl transferase (GGT), glucose, lactate dehydrogenase (LDH), phosphorus, potassium, sodium, total bilirubin, total calcium, total cholesterol, total protein, triglycerides, and uric acid, according to a previous study [18]. Twelve EQA samples (3 samples × 4 shipments) for each hospital have been provided per year for general chemistry in KEQAS EQA. 

The proportion of results with a standard deviation index (SDI) more than 2.0 for each general chemistry test item was compared between CASE and control groups for each year. The SDI was calculated as the difference between laboratory result and the peer group mean divided by peer group standard deviation (SD) at each sample ($\mathrm{SDI}=\frac{(laboratory\;result-peer\;group\;mean}{peer\;group\;SD}$). Results of more than 2 SDI (95% acceptable criteria) are generally regarded as poor results. Therefore, we assumed the proportion of laboratory results with larger than 2 SDI to be a bad laboratory quality marker (BLQM) for each test item. The peer group consisted of laboratories performing the analysis using the same methods, since it is expected that each method will have a similar matrix-related bias for a given specimen.

As a statistical parameter to assess bias in performance between CASE and control groups, we used the SDI of each laboratory’s results. The geometric mean SDIs for each general chemistry test item between CASE and control groups were compared for each year.

### 2.4. Statistical Analysis

BLQM of CASE and control groups were compared by chi-square tests (CASE vs. CON-1 and CASE vs. CON-2). Mean SDIs among three groups (CASE and two control groups) were compared using ANOVAs, followed by post hoc analysis using Duncan’s multiple range test to control the type I comparison-wise error rate. SDI values were reported as the geometric mean with 95% confidence intervals. *p*-values were based on two-sided comparisons, and *p* < 0.05 was considered statistically significant.

### 2.5. Ethics Statement

The study was approved by the Institutional Review Board of Konkuk University, in Korea (approval number: 7001355-201904-E-092). 

## 3. Results

### 3.1. General Characteristics of Study Subjects

The general characteristics of study subjects are shown in Table 1.

### 3.2. Proportion of Results of More than 2 SDI (BLQM) in General Chemistry Tests

**Figure 1:**
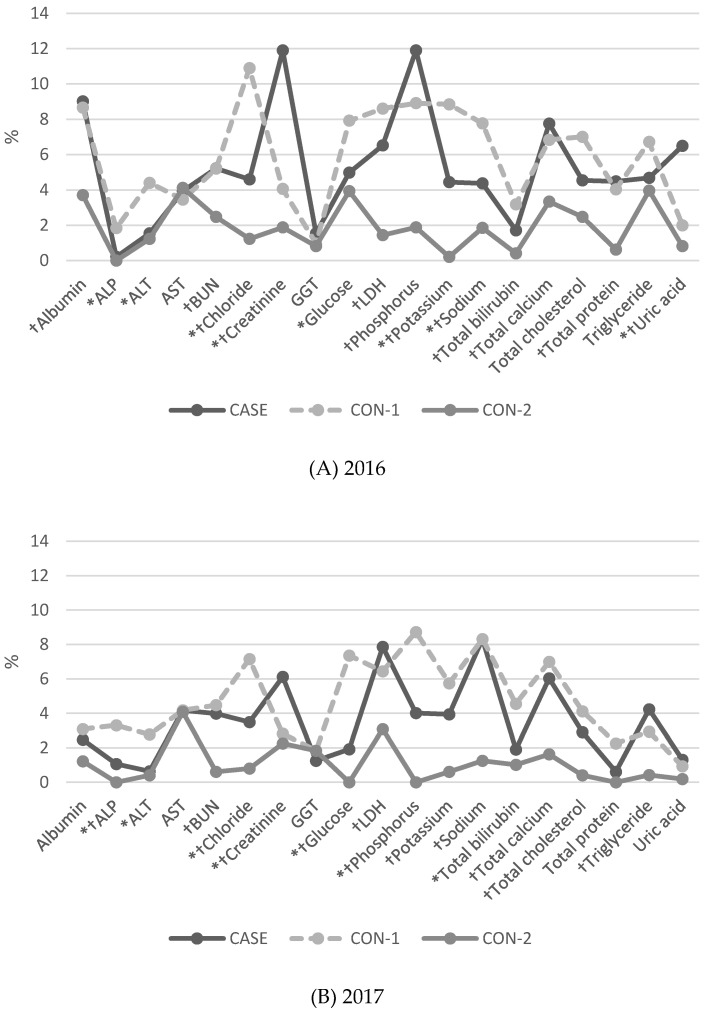

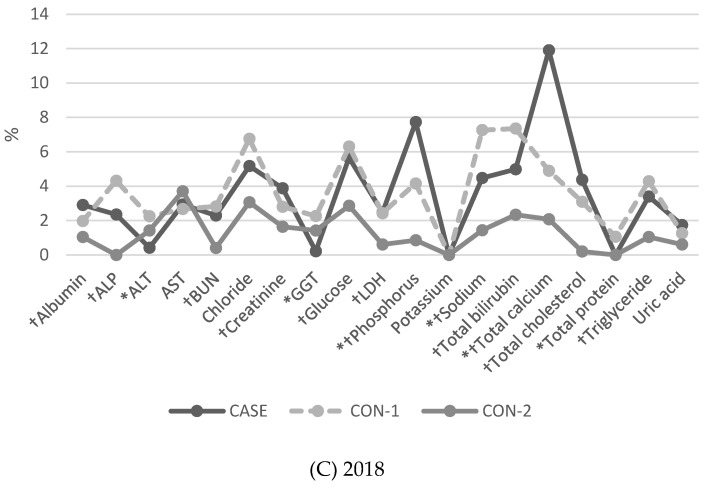
The proportion (%) of general chemistry results of more than 2 standard deviation indexes (SDIs) of Korean Association of External Quality Assessment Service (KEQAS) external quality assessment (EQA) over three years: (**A**) 2016, (**B**) 2017, (**C**) 2018. Abbreviation: CON-1, control 1; CON-2, control 2. * *p* value by chi-square test was lower than 0.05 between CASE and CON-1. ^†^ *p* value by chi-square test was lower than 0.05 between CASE and CON-2. ALP: alkaline phosphatase, ALT: alanine aminotransferase; AST: aspartate transaminase; BUN: blood urea nitrogen; GGT: γ-glutamyl transferase; LDH: lactate dehydrogenase.

BLQMs differed among groups, according to test items or year. The BLQMs of CASE were lower than those of CON-1 for more than two years in ALP, ALT, chloride, glucose, sodium, and total protein, and higher for creatinine. The BLQMs of CASE were higher than those of CON-2 for more than two years in ALP, chloride, creatinine, glucose, LDH, phosphorus, potassium, sodium, total calcium, total cholesterol, triglyceride, and uric acid. There were no test items for which the BLQMs of CASE were lower than those of CON-2 (Figure 1).

### 3.3. Differences in Mean SDIs for General Chemistry Tests

Mean SDIs of general chemistry tests differed among groups, according to test items and year. Mean SDIs in the CASE group were higher than those of CON-1 for ALP, total calcium, and uric acid, but lower for sodium. For total calcium and total protein, mean SDIs were higher in the CASE group than those for CON-2; however, ALP and ALT were lower than those for CON-2 (Table 2).

## 4. Discussion

In this study, we evaluated the effects of pilot NDRGs on laboratory test quality by analyzing general chemistry test data from KEQAS from 2016 to 2018. To the best of our knowledge, this is the first report on this issue. There were some significant differences but in inconsistent directions; that is, some tests were higher quality in the CASE group, while other tests were higher quality in the CON-1 group. 

By implementing DRGs, hospitals lowered laboratory operating costs by limiting laboratory growth and development [19], since costs became the biggest problem for hospital laboratories [20]. As a result, diagnostic tests requiring high operating costs such as complicated or new tests would be avoided [20], and efforts to maintain testing quality were predicted to decrease. However, in this study, we could not find any worsening in laboratory test quality, at least in terms of EQA data, after NDRGs were introduced. These findings should be very cautiously interpreted, however, because this does not necessarily mean that NDRG had no adverse effect on the laboratory test quality. The reimbursement scheme of the current pilot NDRG model is unique, as NDRG participating institutions were reimbursed commensurate with FFS cost, by the “Adjustment Factor” for each institution [10]. Therefore the current pilot NDRG did not encourage healthcare providers to cut unnecessary procedures/tests for cost containment [10]. 

Our findings could also be due to the characteristics of the study population. The majority of the CASE group were public hospitals. Public hospitals have many differences in purpose, operations, and patient demographics compared with private hospitals [11]. Public hospitals are less sensitive to profitability and tend to put more importance on the fulfillment of their mission than on efficiency of care [11]. The characteristics of public hospitals and private hospitals may have caused selection bias that impaired the study’s intrinsic validity. However, that bias was impossible to avoid because there were no private hospitals that participated in NDRG during the evaluation period. In the future, it would be helpful to re-investigate this issue with private hospitals, which may be more sensitive to and dependent upon profits.

Further, EQA data may not be a sensitive enough indicator reflecting laboratory quality changes after the new payment system was introduced. Although EQA is widely used for monitoring medical laboratory performance [16], it reflects quality only at specific points in time, not the laboratory’s overall quality. Additionally, quality improvement in Korea has already reached a fairly high level, due to decades-long efforts in EQA participation.

Last, the study did not fully control for environmental factors other than hospital size. For example, test quality may be positively affected by the laboratory accreditation program participation and ability of working clinical pathologists [18]. As shown in Figure 1, we found that the proportion of bad-quality test results were definitely lower in tertiary hospitals (CON-2) than in the CASE group. These findings may be due to the effects of laboratory accreditation. Jang et al. [18] reported that hospitals with laboratory accreditation showed significantly better results over four years for all general chemistry tests (*p* < 0.0001) compared to hospitals without accreditation. The researchers stressed the need to establish a laboratory accreditation program to standardize diagnostic testing. Further research on the effects of NDRGs is required, using various metrics that can measure overall quality of laboratory services.

Laboratory Medicine Foundation (LMF) accreditation participation shows a low proportion (40.1%) in the CASE group and consistently high proportions in the CON-1 (69.0%) and CON-2 (100%) groups (Table 1). Laboratory accreditation is a good tool for monitoring and improving the total quality of a laboratory. Participation in accreditation is burdensome, however, because it requires time, effort, and money. Therefore, regulations or benefits should encourage participation. In Korea, participants found some incentives (“Comprehensive Verification Fee”) under the FFS system [21] but cannot receive them under NDRGs. Perhaps that is why the participation rate in the CASE group is low.

Laboratory participation in quality assurance systems, such as laboratory accreditation and EQA, may contribute to reducing false-positive or -negative results and unnecessary test requests, thus ensuring patient safety and improving cost-effectiveness [22]. Laboratory quality improvement must be considered in any reimbursement system, and this should be true also in the current and future NDRG model. We suggest that adequate quality indicators be developed and included in NDRG reimbursement schemes to achieve this critical aim. 

## 5. Conclusions

We found some significant differences in the quality of tests between CASE and CON groups, but in inconsistent directions; some tests were higher quality in the CASE group, while other tests were higher quality in the CON-1 group. However, these findings should be interpreted cautiously since the current NDRG program is a pilot program with compensations commensurate to the FFS, and most participants were public hospitals. The effects of NDRGs on laboratory test quality should be re-evaluated after the formal NDRG program is implemented with more private hospitals. We suggest adequate quality indicators be developed and included in the NDRG for laboratory quality improvement.

## Figures and Tables

**Table 1:** General characteristics of study subjects.

	CASE		CON-1		CON-2	
	*N*	(%)	*N*	(%)	*N*	(%)
Total number	42	100	84	100	42	100
NDRG participation time						
at Sep 2012	36		NA		NA	
after Sep 2012	6		NA		NA	
Type of medical institution						
Small-to-medium hospital	9	21.4	11	13.1	0	0.0
General hospital	33	78.6	72	85.7	0	0.0
Tertiary hospital	0	0.0	1	1.2	42	100.0
Number of beds in institution						
<100	4	9.5	8	9.5	0	0.0
100–500	33	78.6	66	78.6	0	0.0
≥500	5	11.9	10	11.9	42	100.0
LMF accreditation ^†^						
2016	17	40.5	58	69.0	42	100.0
2017	18	42.9	58	69.0	42	100.0
2018	18	42.9	58	69.0	42	100.0

Abbreviation: *N*, number; CON-1, control 1; CON-2, control 2; NDRG, new diagnosis-related group; LMF, Laboratory Medicine Foundation. CON-1 and CON-2 subjects were selected among the NDRG non-participating hospitals for the EQA result analysis period (2016–2018). CON-1 subjects were matched according to the number of beds in NDRG participating hospitals and CON-2 subjects were selected as the tertiary hospitals for reference comparison. ^†^ LMF accreditation is a laboratory accreditation program through external on-site inspection by LMF as an independent third party every one or two years. This shows the low proportion (40.1%) in CASE group and consistent high proportion in CON-1 (69.0%) and CON-2 (100%) groups.

**Table 2:** Mean SDIs among three groups for general chemistry tests of KEQAS EQA over three years.

Test Item	Year	SDI (95% CI)			*p*-Value (ANOVA)	Multiple Comparison (Duncan)
		CASE	CON-1	CON-2		
Albumin	2016	0.59 (0.53–0.65)	0.69 (0.64–0.73)	0.62 (0.57–0.68)	0.0161	(CASE, CON-1) *
	2017	0.50 (0.45–0.56)	0.46 (0.42–0.50)	0.50 (0.45–0.55)	0.3529	
	2018	0.44 (0.37–0.51)	0.40 (0.36–0.44)	0.42 (0.37–0.49)	0.5677	
ALP	2016	0.61 (0.56–0.67)	0.59 (0.55–0.63)	0.80 (0.74–0.86)	<0.0001	(CASE, CON-2) (CON-1, CON-2)
	2017	0.62 (0.57–0.68)	0.58 (0.54–0.63)	0.77 (0.71–0.84)	<0.0001	(CASE, CON-2) (CON-1, CON-2)
	2018	0.72 (0.66–0.79)	0.63 (0.59–0.68)	0.86 (0.79–0.94)	<0.0001	(CASE, CON-1) (CASE, CON-2) (CON-1, CON-2)
ALT	2016	0.41 (0.37–0.46)	0.47 (0.43–0.50)	0.52 (0.47–0.57)	0.01	(CASE, CON-2)
	2017	0.31 (0.27–0.36)	0.33 (0.30–0.37)	0.32 (0.28–0.37)	0.7598	
	2018	0.43 (0.38–0.48)	0.46 (0.43–0.50)	0.50 (0.45–0.55)	0.1296	(CASE, CON-2)
AST	2016	0.50 (0.45–0.55)	0.55 (0.52–0.59)	0.65 (0.59–0.71)	0.0004	(CASE, CON-2) (CON-1, CON-2)
	2017	0.34 (0.29–0.40)	0.41 (0.38–0.46)	0.47 (0.41–0.54)	0.0079	(CASE, CON-1) (CASE, CON-2)
	2018	0.41 (0.36–0.47)	0.45 (0.42–0.49)	0.47 (0.41–0.53)	0.3206	
BUN	2016	0.51 (0.47–0.56)	0.50 (0.47–0.54)	0.38 (0.34–0.42)	<0.0001	(CASE, CON-2) (CON-1, CON-2)
	2017	0.41 (0.36–0.46)	0.42 (0.39–0.46)	0.31 (0.28–0.35)	0.0002	(CASE, CON-2) (CON-1, CON-2)
	2018	0.42 (0.37–0.46)	0.43 (0.40–0.47)	0.40 (0.36–0.44)	0.4678	
Chloride	2016	0.54 (0.50–0.59)	0.63 (0.59–0.67)	0.45 (0.42–0.49)	<0.0001	(CASE, CON-1) (CASE, CON-2) (CON-1, CON-2)
	2017	0.51 (0.45–0.57)	0.51 (0.47–0.56)	0.52 (0.47–0.58)	0.9161	
	2018	0.38 (0.33–0.45)	0.47 (0.42–0.52)	0.43 (0.38–0.49)	0.099	
Creatinine	2016	0.62 (0.55–0.69)	0.50 (0.46–0.53)	0.38 (0.34–0.42)	<0.0001	(CASE, CON-1) (CASE, CON-2) (CON-1, CON-2)
	2017	0.50 (0.45–0.55)	0.40 (0.38–0.43)	0.36 (0.33–0.41)	0.0002	(CASE, CON-1) (CASE, CON-2)
	2018	0.45 (0.41–0.50)	0.41 (0.39–0.45)	0.38 (0.34–0.43)	0.1	
GGT	2016	0.52 (0.47–0.58)	0.53 (0.49–0.57)	0.44 (0.39–0.49)	0.0126	(CASE, CON-2) (CON-1, CON-2)
	2017	0.41 (0.36–0.47)	0.46 (0.42–0.50)	0.48 (0.43–0.53)	0.1426	
	2018	0.42 (0.37–0.47)	0.43 (0.39–0.47)	0.42 (0.36–0.48)	0.9065	
Glucose	2016	0.38 (0.34–0.43)	0.51 (0.48–0.56)	0.40 (0.36–0.44)	<0.0001	(CASE, CON-1) (CON-1, CON-2)
	2017	0.36 (0.32–0.42)	0.49 (0.45–0.54)	0.33 (0.29–0.38)	<0.0001	(CASE, CON-1) (CON-1, CON-2)
	2018	0.36 (0.31–0.41)	0.38 (0.34–0.43)	0.30 (0.25–0.35)	0.0268	(CON-1, CON-2)
LDH	2016	0.50 (0.45–0.56)	0.55 (0.50–0.59)	0.37 (0.33–0.42)	<0.0001	(CASE, CON-2) (CON-1, CON-2)
	2017	0.45 (0.38–0.52)	0.40 (0.35–0.45)	0.37 (0.32–0.43)	0.2316	
	2018	0.58 (0.52–0.66)	0.59 (0.54–0.64)	0.57 (0.52–0.62)	0.9349	
Phosphorus	2016	0.56 (0.49–0.64)	0.45 (0.41–0.50)	0.34 (0.29–0.39)	<0.0001	(CASE, CON-1) (CASE, CON-2) (CON-1, CON-2)
	2017	0.33 (0.27–0.40)	0.41 (0.36–0.46)	0.34 (0.30–0.40)	0.0641	
	2018	0.42 (0.36–0.51)	0.35 (0.31–0.40)	0.33 (0.28–0.38)	0.0828	
Potassium	2016	0.52 (0.47–0.57)	0.57 (0.52–0.62)	0.50 (0.46–0.54)	0.0605	
	2017	0.19 (0.16–0.24)	0.22 (0.19–0.25)	0.16 (0.13–0.20)	0.0652	
	2018	0.31 (0.26–0.38)	0.34 (0.30–0.39)	0.31 (0.26–0.36)	0.5434	
Sodium	2016	0.51 (0.45–0.57)	0.54 (0.50–0.59)	0.44 (0.40–0.49)	0.0252	(CASE, CON-2)
	2017	0.38 (0.32–0.46)	0.44 (0.39–0.49)	0.28 (0.23–0.33)	<0.0001	(CASE, CON-2)
	2018	0.27 (0.23–0.32)	0.37 (0.33–0.42)	0.37 (0.32–0.43)	0.008	(CASE, CON-1)
Total bilirubin	2016	0.56 (0.51–0.60)	0.68 (0.64–0.72)	0.60 (0.55–0.65)	0.0004	(CASE, CON-1) (CON-1, CON-2)
	2017	0.63 (0.58–0.69)	0.61 (0.57–0.65)	0.56 (0.51–0.62)	0.1957	
	2018	0.61 (0.56–0.68)	0.59 (0.55–0.63)	0.67 (0.62–0.73)	0.0998	
Total calcium	2016	0.59 (0.53–0.66)	0.46 (0.42–0.50)	0.42 (0.37–0.47)	<0.0001	(CASE, CON-1) (CASE, CON-2)
	2017	0.46 (0.36–0.53)	0.40 (0.36–0.44)	0.27 (0.23–0.32)	<0.0001	(CASE, CON-2) (CON-1, CON-2)
	2018	0.52 (0.46–0.59)	0.40 (0.37–0.44)	0.33 (0.29–0.38)	<0.0001	(CASE, CON-1) (CASE, CON-2) (CON-1, CON-2)
Total cholesterol	2016	0.42 (0.37–0.47)	0.53 (0.49–0.58)	0.39 (0.35–0.44)	<0.0001	(CASE, CON-1) (CON-1, CON-2)
	2017	0.39 (0.34–0.45)	0.49 (0.45–0.53)	0.39 (0.35–0.44)	0.0009	(CASE, CON-1) (CON-1, CON-2)
	2018	0.44 (0.40–0.50)	0.47 (0.44–0.51)	0.46 (0.41–0.51)	0.6273	
Total protein	2016	0.35 (0.30-0.42)	0.32 (0.29-0.37)	0.24 (0.20–0.29)	0.0062	(CASE, CON-2) (CON-1, CON-2)
	2017	0.37 (0.33–0.42)	0.45 (0.41–0.48)	0.33 (0.29–0.37)	<0.0001	(CASE, CON-1) (CON-1, CON-2)
	2018	0.31 (0.27–0.36)	0.34 (0.31–0.38)	0.24 (0.20–0.28)	0.0006	(CASE, CON-2)
Triglyceride	2016	0.44 (0.39–0.48)	0.51 (0.48–0.55)	0.39 (0.35–0.44)	0.0001	(CASE, CON-1) (CON-1, CON-2)
	2017	0.40 (0.35–0.46)	0.38 (0.34–0.42)	0.31 (0.27–0.35)	0.0193	(CASE, CON-2) (CON-1, CON-2)
	2018	0.38 (0.33–0.43)	0.42 (0.38–0.46)	0.33 (0.29–0.37)	0.0057	(CON-1, CON-2)
Uric acid	2016	0.49 (0.43–0.55)	0.45 (0.41–0.49)	0.45 (0.39–0.51)	0.6028	
	2017	0.35 (0.31–0.41)	0.42 (0.38–0.46)	0.37 (0.33–0.42)	0.0916	
	2018	0.43 (0.37–0.49)	0.32 (0.28–0.36)	0.38 (0.33–0.44)	0.0056	(CASE, CON-1)

* The two groups in parentheses mean there is a statistically significant difference in mean SDI.

## References

[1] Kim K.H., Lee S.C., Lee S.K., Choi B.J., Jeong W., Kim S.J. (2016). Does Korea’s current diagnosis-related group-based reimbursement system appropriately classify appendectomy patients?. Ann. Surg. Treat. Res..

[2] Vecellio E., Li L., Xiong J., Georgiou A., Eigenstetter A., Gibson-Roy C., Cobain T., Golding M., Wilson R., Lindeman R. (2015). Examination of Variation in Hospital Pathology Investigations by Diagnosis-Related Groups and Associations with Outcomes and Costs.

[3] Geissler A., Scheller-Kreinsen D., Quentin W., Euro D.R.G.G. (2012). Do diagnosis-related groups appropriately explain variations in costs and length of stay of hip replacement? A comparative assessment of DRG systems across 10 European countries. Health Econ..

[4] Fassler M., Wild V., Clarinval C., Tschopp A., Faehnrich J.A., Biller-Andorno N. (2015). Impact of the DRG-based reimbursement system on patient care and professional practise: Perspectives of Swiss hospital physicians. Swiss Med. Wkly..

[5] Choi J.W., Kim S.J., Park H.K., Jang S.I., Kim T.H., Park E.C. (2019). Effects of a mandatory DRG payment system in South Korea: Analysis of multi-year nationwide hospital claims data. BMC Health Serv Res..

[6] Hu W.Y., Yeh C.F., Shiao A.S., Tu T.Y. (2015). Effects of diagnosis-related group payment on health-care provider behaviors: A consecutive three-period study. J. Chin. Med. Assoc..

[7] Jung Y.W., Pak H., Lee I., Kim E.H. (2018). The Effect of Diagnosis-Related Group Payment System on Quality of Care in the Field of Obstetrics and Gynecology among Korean Tertiary Hospitals. Yonsei Med. J..

[8] Kim S.J., Han K.T., Kim W., Kim S.J., Park E.C. (2018). Early Impact on Outpatients of Mandatory Adoption of the Diagnosis-Related Group-Based Reimbursement System in Korea on Use of Outpatient Care: Differences in Medical Utilization and Presurgery Examination. Health Serv Res..

[9] Choi J.K., Kim S.H., Shin D.G., Kang J.G. (2017). The Effect of Reform of New-Diagnosis Related Groups (KDRGs) on Accuracy of Payment. Health Policy Manag..

[10] Kim M. (2017). Status and Future Challenges of the New DRG system. Policy Trends.

[11] Kim G.-D., Park J.H. (2018). Effects of New Diagnosis-Related Group-Based Payment on Efficiency of Public Hospitals. Korean J. Public Adm..

[12] Ngo A., Gandhi P., Miller W.G. (2017). Frequency that Laboratory Tests Influence Medical Decisions. J. Appl. Lab. Med..

[13] World Health Organizsation (2016). WHO Manual for Organizing a Nati Onal External Quality Assessment Programme for Health Laboratories and Other Testing Sites.

[14] Korean Ministry of Health and Welfare (2017). Details on the application criteria and methods of healthcare benefit. Korean Ministry of Health and Welfare Notification 2017-111.

[15] Korean Medical Service Act. https://elaw.klri.re.kr/.

[16] Krleza J.L., Celap I., Tanaskovic J.V. (2017). External Quality Assessment in Croatia: Problems, challenges, and specific circumstances. Biochem Med..

[17] Kim H., Kim S., Yun Y.M., Um T.H., Chang J., Lee K.S., Chun S., Cho K.D., Han T.H. (2020). Status of Quality Control for Laboratory Tests of Medical Institutions in Korea: Analysis of 10 Years of Data on External Quality Assessment Participation. Healthcare.

[18] Jang M.A., Yoon Y.A., Song J., Kim J.H., Min W.K., Lee J.S., Lee Y.W., Lee Y.K. (2017). Effect of Accreditation on Accuracy of Diagnostic Tests in Medical Laboratories. Ann. Lab. Med..

[19] Takemura Y., Beck J.R. (1999). The effects of a fixed-fee reimbursement system introduced by the Federal Government on laboratory testing in the United States. Rinsho Byori.

[20] Shimetani N. (2004). Medical reimbursement for diagnostic tests. Expert Rev. Pharm. Outcomes Res..

[21] Department of Medical Insurance Benefits Development (2003). Details of Health Insurance Care Benefits Expenses.

[22] Jun J.K., Sung N.Y., Song S.H., Hong S., Jang M.A., Song J., Kim J.H., Min W.K., Lee Y.K. (2018). Budget Impact of the Accreditation Program for Clinical Laboratories on Colorectal Cancer Screening via Fecal Immunochemical Testing: Results from the National Cancer Screening Program in Korea. Ann. Lab. Med..

